# The Deficiency of Hypusinated eIF5A Decreases the Putrescine/Spermidine Ratio and Inhibits +1 Programmed Ribosomal Frameshifting during the Translation of Ty1 Retrotransposon in *Saccharomyces cerevisiae*

**DOI:** 10.3390/ijms25031766

**Published:** 2024-02-01

**Authors:** Yu Xiao, Ruanlin Wang, Xiaxia Han, Wei Wang, Aihua Liang

**Affiliations:** 1 Key Laboratory of Chemical Biology and Molecular Engineering of Ministry of Education, Institute of Biotechnology, Shanxi University, Taiyuan 030006, China; 202013002003@email.sxu.edu.cn (Y.X.); rlwang@sxu.edu.cn (R.W.); hxx20208@163.com (X.H.); 2 Shanxi Key Laboratory of Biotechnology, Taiyuan 030006, China

**Keywords:** *Saccharomyces cerevisiae*, programmed ribosomal frameshifting, eIF5A, Ty1 retrotransposon

## Abstract

Programmed ribosomal frameshifting (PRF) exists in all branches of life that regulate gene expression at the translational level. The eukaryotic translation initiation factor 5A (eIF5A) is a highly conserved protein essential in all eukaryotes. It is identified initially as an initiation factor and functions broadly in translation elongation and termination. The hypusination of eIF5A is specifically required for +1 PRF at the shifty site derived from the *ornithine decarboxylase antizyme 1* (*OAZ1*) in *Saccharomyces cerevisiae*. However, whether the regulation of +1 PRF by yeast eIF5A is universal remains unknown. Here, we found that Sc-eIF5A depletion decreased the putrescine/spermidine ratio. The re-introduction of Sc-eIF5A in yeast eIF5A mutants recovered the putrescine/spermidine ratio. In addition, the Sc-eIF5A depletion decreases +1 PRF during the decoding of *Ty1* retrotransposon mRNA, but has no effect on −1 PRF during the decoding of *L-A* virus mRNA. The re-introduction of Sc-eIF5A in yeast eIF5A mutants restored the +1 PRF rate of Ty1. The inhibition of the hypusine modification of yeast eIF5A by GC7 treatment or by mutating the hypusination site Lys to Arg caused decreases of +1 PRF rates in the *Ty1* retrotransposon. Furthermore, mutational studies of the *Ty1* frameshifting element support a model where the efficient removal of ribosomal subunits at the first *Ty1* frame 0 stop codon is required for the frameshifting of trailing ribosomes. This dependency is likely due to the unique position of the frame 0 stop codon distance from the slippery sequence of *Ty1*. The results showed that eIF5A is a *trans*-regulator of +1 PRF for *Ty1* retrotransposon and could function universally in yeast.

## 1. Introduction

Programmed ribosomal frameshifting (PRF) is a recoding event by which the translating ribosome switches from the initial (0) reading frame to the +1 or −1 reading frame at a specific position, and then continues its translation [1,2]. Unlike frameshift mutations, PRF can be regulated by cis-acting elements and *trans*-acting factors, and has important biological functions [3]. This phenomenon was first discovered in viruses [4]. The efficiency of PRF determines the stoichiometric ratio between viral Gag (structural) and Gag–Pol fusion (enzymatic) proteins, and it has been demonstrated in many different viral systems that viral particle assembly and propagation are inhibited when changing PRF efficiencies [5,6,7,8,9,10]. The PRF is widespread and likely exists from bacteria to higher eukaryotes [11,12,13].

The efficiency of PRF is regulated not only by cis-regulatory elements in the mRNA but also by *trans*-acting factors, such as tRNAs [14,15,16], polyamines [17,18], antibiotics [19,20] and proteins [11,21]. In yeast, the cis-regulatory elements are the slippery sequence and stimulatory RNA secondary structures. The stimulatory RNA secondary structures act as a roadblock for rapid translation. The slippery sequence and the induction of a ribosomal pause are required to promote efficient frameshifting [22]. Currently, it has been reported that *trans*-acting factors modulate PRF in yeast. A rare tRNA-Arg (CCU) that regulates *Ty1* element ribosomal frameshifting is essential for Ty1 retrotransposition [14], and high polyamine levels that regulate *OAZ1* element ribosomal frameshifting are essential for OAZ1 [23].

The eukaryotic translation initiation factor 5A (eIF5A) is essential for cell viability and is highly conserved in all eukaryotes [24]. It is the only protein known to carry hypusination, an unusual post-translational modification [24]. Hypusine is a modified lysine residue found in eIF5A that is required for its activity. Although the hydroxylation of desoxyhypusinated-eIF5A, the last step of hypusination, is not essential in *Saccharomyces cerevisiae*, this complete post-translational modification of eIF5A is strictly required for its function in higher eukaryotes [25,26]. eIF5A was originally thought to stimulate the formation of the first peptide bond during the translation initiation phase [24]. Then, its involvement in translating polyproline-containing proteins was discovered [27]. Recent studies based on the ribosome profile data suggested that eIF5A works more generally at many ribosome-stalled sites [28,29]. eIF5A binds to the ribosomal E site to promote the peptide bond formation of sterically unfavorable amino acid combinations and plays a critical role in peptidyl-tRNA hydrolysis after stop codon recognition. Furthermore, the hypusination of eIF5A is specifically required for +1 PRF at the shifty site derived from the *OAZ1* in *S. cerevisiae* [30]. In addition, it is also associated with the synthesis of proteins involved in polyamine synthesis and transport [31,32,33,34].

In addition to *OAZ1*, the L-A virus and Ty1 retrotransposon of the yeast *S. cerevisiae* have been especially useful in characterizing the molecular genetics and biochemistry of PRF [22]. A −1 PRF event is responsible for producing the Gag–Pol fusion protein of the L-A virus of yeast [35,36]. The 5′ *gag* gene encodes the major coat protein, and the 3′ *pol* gene encodes a multifunctional protein domain, which includes the RNA-dependent RNA polymerase and a domain required for viral RNA packaging [22]. The promotion of efficient −1 PRF in the L-A virus of yeast requires a special sequence, X XXY YYZ (the 0-frame is indicated by spaces) called the ‘slippery site’ [22]. The simultaneous slippage of ribosome-bound A- and P-site tRNAs by one base in the 5′ direction still leaves their non-wobble bases correctly paired in the new reading frame [22]. A second promoting element, usually an mRNA pseudoknot, is located immediately 3′ to the slippery site [22]. It is thought that the role of the mRNA pseudoknot is to induce elongating ribosomes to pause over the slippery site [22]. Furthermore, a +1 PRF event, directed by a heptanucleotide sequence CUU AGG C, is responsible for producing the Gag–Pol fusion protein of the yeast retrotransposon Ty1 [22,37]. Although both +1 and −1 ribosomal frameshifting occurs at heptameric “slippery sites”, the nature of these sites is entirely different. Unlike −1 ribosomal frameshifting, the simultaneous slippage of ribosome-bound A- and P-site tRNAs from the 0-frame to the +1 frame would not allow their non-wobble bases to repair. Also, in −1 ribosomal frameshifting, the downstream sequence required to promote efficient frameshifting is the mRNA pseudoknot. Although a potential pseudoknot structure can be inferred in *Ty1*, the structure is not required [22]. In addition, the outcome of −1 PRF of *CTS2* was also identified in the yeast, which is predicted to direct ribosomes to a premature termination signal [38].

To explore the function of Sc-eIF5A on +1 PRF and −1 PRF, two eIF5A temperature-sensitive yeast strains, *tif51A-1* and *tif51A-3*, were used in the study. The loss of Sc-eIF5A reduced the putrescine/spermidine ratio. The re-introduction of Sc-eIF5A in yeast mutants recovered the putrescine/spermidine ratio. Moreover, Sc-eIF5A depletion decreases +1 PRF in *Ty1*, but has no effect on −1 PRF in *L-A*. The re-introduction of Sc-eIF5A in yeast eIF5A mutants restored the efficiency of +1 PRF in *Ty1*. In addition, the impaired hypusine modification of yeast eIF5A by GC7 treatment or by mutating the hypusination site leads to decreases in +1 PRF in *Ty1*. Mutational studies of the *Ty1* frameshifting element suggested a model in which the efficient removal of a post-termination ribosome on the *Ty1* frame 0 stop codon by Sc-eIF5A is necessary for a trailing ribosome to stall at the slippery sequence and undergo frameshifting. These findings showed that eIF5A-ployamine feedback regulation was essential for +1 PRF in yeast.

## 2. Results

### 2.1. Sc-eIF5A Depletion Decreases the Putrescine/Spermidine Ratio

**Figure 1:**
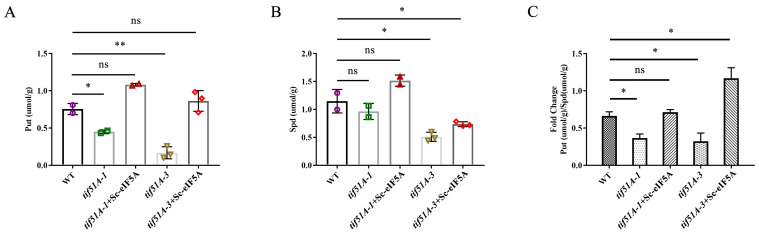
The eIF5A depletion decreases the putrescine and spermidine levels. (**A**) HPLC analysis of the putrescine levels of WT, *tif51A-1*, *tif51A-3*, Sc-eIF5A-complemented *tif51A-1* and Sc-eIF5A-complemented *tif51A-3* strains. (**B**) HPLC analysis of the spermidine levels of WT, *tif51A-1*, *tif51A-3*, Sc-eIF5A-complemented *tif51A-1* and Sc-eIF5A-complemented *tif51A-3* strains. (**C**) The putrescine/spermidine ratios of WT, *tif51A-1*, *tif51A-3*, Sc-eIF5A-complemented *tif51A-1* and Sc-eIF5A-complemented *tif51A-3* strains. Error bars denote SD. And individual replicates are plotted with a symbol. Different symbols represent different groups of replicates. * *p* < 0.05, ** *p* < 0.01, ns, not significant (Student’s two-tailed *t* test, *n* = 3, assayed in duplicate).

The hypusination of eIF5A is a multi-step process, during which a 4-aminobutyl moiety, derived from spermidine, is transferred to a specific lysine residue of eIF5A (K51 in yeast Hyp2 or Anb1). However, high levels of polyamines were shown to stimulate ribosomal frameshifting during the decoding of *OAZ1* mRNA and *Ty1* mRNA in yeast, respectively [18,23]. Furthermore, eIF5A is also associated with the synthesis of proteins involved in polyamine synthesis and transport [31,32,33,34]. The polyamine levels were monitored in eIF5A-deficient and eIF5A-complementary strains by HPLC. The putrescine level was lower in the *tif51A-1* and *tif51A-3* strains than in the WT strain (Figure 1A). After the re-introduction of WT Sc-eIF5A in the *tif51A-1* and *tif51A-3* strains, the putrescine content was restored to that of the WT strain (Figure 1A,B). However, in the absence or complementation of Sc-eIF5A, the spermidine level did not change significantly in the *tif51A-1* strain compared to that in the WT. Nevertheless, the spermidine level was significantly decreased in the *tif51A-3* strain than in the WT. The spermidine level was increased after Sc-eIF5A complementation. Given the known importance of the putrescine/spermidine ratio in modulating ribosomal frameshifting in yeast [18], the putrescine/spermidine ratios in WT, eIF5A-deficient and eIF5A-complementary strains were calculated. The putrescine/spermidine ratios from the *tif51A-1* and *tif51A-3* strains were both much lower than the WT strain. After the re-introduction of WT Sc-eIF5A in the *tif51A-1* and *tif51A-3* strains, however, the putrescine/spermidine ratios increased significantly, and even exceeded the level of the WT strain (Figure 1C).

### 2.2. Sc-eIF5A Depletion Decreases +1 Programmed Ribosomal Frameshifting Efficiency

**Figure 2:**
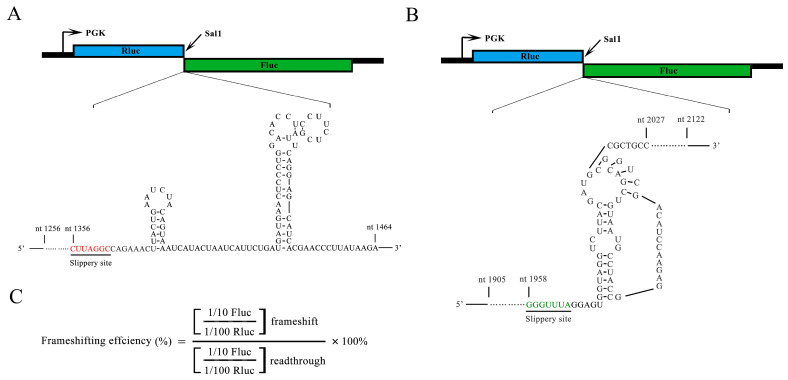
Secondary structural representation of the PRF signals used in this study. Graphical representation of the pDB722-Ty1 (**A**) and pDB722-L-A (**B**). (**C**) The formula for calculating the +1 PRF efficiency. Red and green underlined text represent the slippery sequence of *Ty1* and *L-A*, respectively.

To investigate the influence of Sc-eIF5A on +1 PRF and −1 PRF, three PRF reporter constructs, pDB722-Ty1, pDB722-CTS2, and pDB722-L-A, were generated (Figure 2A,B and Figure S1A). The frameshift reporter plasmid is preceded by a Renilla luciferase gene and followed by the complete PRF element and a sequence encoding a Firefly luciferase. The Firefly luciferase production depends on +1 or −1 PRF, while Renilla luciferase serves as the internal control. An in-frame control reporter, pDB722, in which the Fluc is in the same reading frame as Rluc, provides baseline data. Frameshift efficiencies were calculated by dividing the Fluc/Rluc activity ratio generated from the frameshift reporter by the same ratio generated from the in-frame control reporter (Figure 2C).

**Figure 3:**
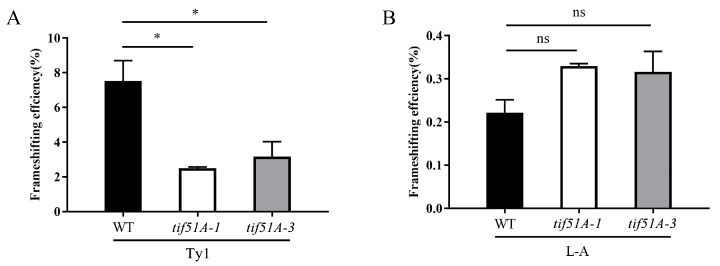
Depletions of Sc-eIF5A in the *tif51A-1* and *tif51A-3* strains decrease +1, but not −1, PRF, respectively. (**A**) Dual-luciferase reporter plasmids containing Fluc and Rluc coding regions separated by the +1 PRF signal from the yeast Ty1 retrotransposon, or (**B**) the −1 PRF signal from the yeast L-A virus, or the 0-frame control were introduced into WT, *tif51A-1* and *tif51A-3* strains. PRF efficiencies (%) were calculated by dividing the ratio of Fluc to Rluc obtained with the reporter versus the 0-frame control plasmid. Error bars denote SD. * *p* < 0.05, ns, not significant (Student’s two-tailed *t* test, *n* = 3, assayed in duplicate).

Since eIF5A is an essential protein in yeast, two eIF5A temperature-sensitive strains, *tif51A-1* and *tif51A-3*, were used [39]. The two eIF5A mutant cells were incubated at 37 °C for 5 h, which led to a significant reduction in eIF5A. Three PRF reporter plasmids (Figure 2A,B and Figure S1A), or an in-frame control reporter plasmid, were transformed into the WT strain and two eIF5A yeast mutants, respectively. Then, after these yeast strains were incubated at 37 °C for 5 h, Fluc and Rluc activities were assayed. The WT strain containing a *Ty1* PRF construct exhibited a frameshifting rate of approximately 7.52%, +1 PRF rates from the *tif51A-1* and *tif51A-3* strains containing the *Ty1* PRF construct were approximately 2.5% and 3.17%, respectively (Figure 3A). The −1 PRF efficiencies from the *tif51A-1* and *tif51A-3* strains containing the *CTS2* PRF construct were decreased by 1.82% and 3.24%, respectively (Figure S1B). However, the −1 PRF efficiencies from the *tif51A-1* and *tif51A-3* strains containing the *L-A* PRF construct were similar to the WT strain containing the *L-A* PRF construct (Figure 3B). These results indicated that Sc-eIF5A promotes the translation of the +1 PRF gene of *Ty1* and the −1 PRF gene of *CTS2*, but does not influence the translation of the −1 PRF gene of *L-A*.

**Figure 4:**
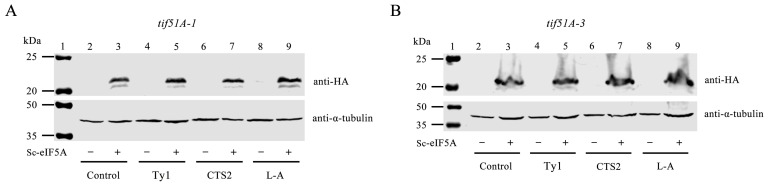
Sc-eIF5A could complement the functional loss of *tif51A-1* and *tif51A-3* mutant strains. (**A**) Expressions of the eIF5A–HA fusion gene in *tif51A-1* strains containing *Ty1*, *CTS2* or *L-A* PRF construct were shown by Western blot analysis, respectively. (**B**) Expressions of the eIF5A–HA fusion gene in tif51A-3 strains containing *Ty1*, *CTS2* or *L-A* PRF construct were shown by Western blot analysis, respectively. “+” and “−” denote that the Sc-eIF5A were transformed or untransformed in the Western blot analysis.

**Figure 5:**
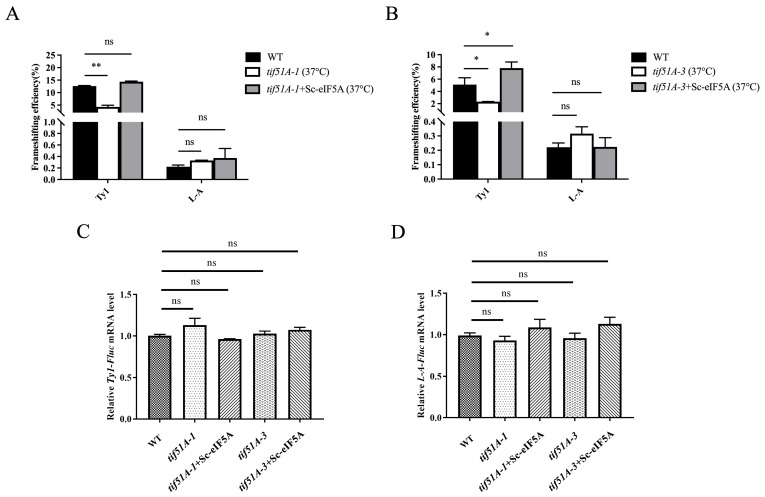
Re-introduction of Sc-eIF5A in the *tif51A-1* and *tif51A-3* strains enhanced Ty1 +1 PRF. (**A**) *tif51A-1* and Sc-eIF5A-complemented *tif51A-1* strains were grown at 37 °C for 5 h. (**B**) *tif51A-3* and Sc-eIF5A-complemented *tif51A-3* strains were grown at 37 °C for 5 h. Dual-luciferase reporter plasmids containing Fluc and Rluc coding regions separated by the +1 PRF signal from the yeast Ty1 retrotransposon, or the −1 PRF signal from the yeast L-A virus, or the 0-frame control were introduced into WT, *tif51A-1* and *tif51A-3* mutant strains. PRF efficiencies (%) were calculated by dividing the ratio of Fluc to Rluc obtained with the reporter versus the 0-frame control plasmid. Results are the average of at least three independent experiments. Relative *Ty1-Fluc* (**C**) and *L-A-Fluc* (**D**) mRNA levels were determined by qPCR and first normalized to actin mRNA. Then, for each panel, measurements were normalized to WT samples. Error bars denote SD. * *p* < 0.05, ** *p* < 0.01, ns, not significant (Student’s two-tailed *t* test, *n* = 3, assayed in duplicate).

To further investigate the effects of Sc-eIF5A on −1 and +1 PRF, the gene encoding yeast HYP2 was cloned into the pRS315 vector. The pRS315-Sc-HYP2 was transformed into *tif51A-1* and *tif51A-3* strains. The Sc-eIF5A-complemented *tif51A-1* and *tif51A-3* strains that expressed Sc-eIF5A-C-HA fusion proteins were obtained (Figure 4A,B). Following the Sc-eIF5A complement, the strains were harvested at 37 °C for 5 h. After the re-introduction of Sc-eIF5A in the *tif51A-1* and *tif51A-3* strains, +1 PRF of *Ty1* was restored to that of the WT strain (Figure 5A,B). Notably, the re-introduction of Sc-eIF5A in the *tif51A-1* and *tif51A-3* strains had minimal effects on −1 PRF at the shifty site of *CTS2* and *L-A* (Figure 5A,B and Figure S1D,E), indicating that Sc-eIF5A promotes the translation of the +1, but not −1 PRF genes.

In order to verify whether the decrease in programmed ribosomal frameshifting efficiency was caused by the decrease in the transcript levels of the fusion genes from constructs, total RNA from the WT, *tif51A-1*, *tif51A-3*, Sc-eIF5A-complemented *tif51A-1* and Sc-eIF5A-complemented *tif51A-3* strains at 37 °C for 5 h, containing pDB722-Ty1, or pDB722-CTS2, or pDB722-L-A, were extracted. And, equal amounts of cDNA were analyzed by qPCR (Figure 5C,D and Figure S1C). There were no significant differences in *Ty1-Fluc*, *CTS2-Fluc*, and *L-A-Fluc* mRNA expression levels for the WT, yeast mutants, and Sc-eIF5A-complemented strains, which indicated that the *Ty1*, *CTS2*, and *L-A* transcriptional rates of yeast mutants were essentially equivalent in the WT strains and Sc-eIF5A-complemented strains.

The reference luciferase (Renilla) activities were dramatically reduced in *S. cerevisiae* transfected with reporters containing the *CTS2* signal compared to the in-frame control (Figures S2C and S3E,G). However, the Renilla luciferase of Ty1 and L-A displayed similar activities for all constructs, respectively (Figures S2A,E and S3A,C,I,K). So, this dual-luciferase-based reporter system is unsuitable for detecting −1 programmed ribosomal frameshifting efficiency during the decoding of *CTS2* mRNA in yeast, which is concordant with previous reports showing that absolute luciferase activities were reduced in HeLa cells transfected with reporters containing the *CCR5* sequence compared to the HIV-1 control and IFC [40,41].

### 2.3. The Hypusine Modification of Sc-eIF5A Influences +1 Programmed Ribosomal Frameshifting Efficiency

**Figure 6:**
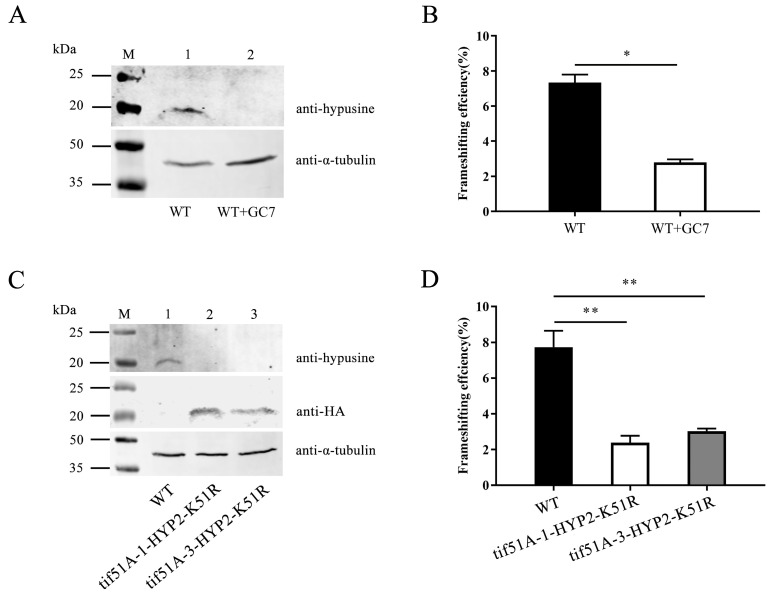
The hypusination depletion of Sc-eIF5A decreases the *Ty1* +1 PRF. (**A**) WT and GC7-treated WT strains were grown at 37 °C for 5 h, and the hypusine levels of Sc-eIF5A in WT and GC7-treated WT strains were shown by Western blot analysis. Cultures contained 1mM GC7. (**B**) WT and GC7-treated WT strains were grown at 37 °C for 5 h, and *Ty1* +1 PRF was detected. Cultures contained 1 mM GC7. (**C**) WT, tif51A-1-HYP2-K51R and tif51A-3-HYP2-K51R strains were grown at 37 °C for 5 h, and the levels of Sc-eIF5A and its hypusine modification in WT, tif51A-1-HYP2-K51R and tif51A-3-HYP2-K51R strains were shown by Western blot analysis, respectively. (**D**) WT, tif51A-1-HYP2-K51R and tif51A-3-HYP2-K51R strains were grown at 37 °C for 5 h, and *Ty1* +1 PRF was detected. Dual-luciferase reporter plasmids containing Fluc and Rluc coding regions separated by the +1 PRF signal from the yeast Ty1 retrotransposon or the 0-frame control were introduced into the WT strain. PRF efficiencies (%) were calculated by dividing the ratio of Fluc to Rluc obtained with the reporter versus the 0-frame control plasmid. Error bars denote SD. * *p* < 0.05, ** *p* < 0.01 (Student’s two-tailed *t* test, *n* = 3, assayed in duplicate).

The hypusine modification in eukaryotes is achieved by the sequential reactions catalyzed by two enzymes: deoxyhypusine synthase (DHS) and deoxyhypusine hydroxylase (DOHH). To investigate whether the hypusine modification is sufficient for the Sc-eIF5A control of +1 PRF, we took advantage of the N1-Guanyl-1,7-diaminoheptane (GC7), a potent inhibitor of DHS. At the treatment of the WT strain with the DHS inhibitor GC7 at 37 °C for 5 h, the hypusine modification of Sc-eIF5A was completely inhibited (Figure 6A and Figure S5A). Ty1 +1 PRF was reduced from 7.34% to 2.79% (Figure 6B), and luciferase values of Ty1 for each experiment were shown in Figure S4A,B.

Further, the unhypusinated Sc-eIF5A^K51R^ was expressed in the *tif51A-1* and *tif51A-3* strains. Western blot analysis indicated that the Sc-eIF5AK51R-C-HA of which the HA tag does not interfere with the hypusination of Sc-eIF5A [42] from the *tif51A-1* and *tif51A-3* strains was produced. The hypusine modification of Sc-eIF5A was completely inhibited (Figure 6C and Figure S5B). In addition, the tif51A-1-HYP2-K51R and tif51A-3-HYP2-K51R strains caused the specific inhibition of +1 PRF at the shifty site of *Ty1* (Figure 6D), and luciferase values of Ty1 for each experiment were shown in Figure S4C,D.

The results suggest that the hypusine modification of Sc-eIF5A plays a crucial role in influencing +1 PRF at the shifty site of *Ty1*.

### 2.4. The Ty1 Frame 0 Stop Codon Position Confers the Dependency of +1 Programmed Ribosomal Frameshifting on Sc-eIF5A

**Figure 7:**
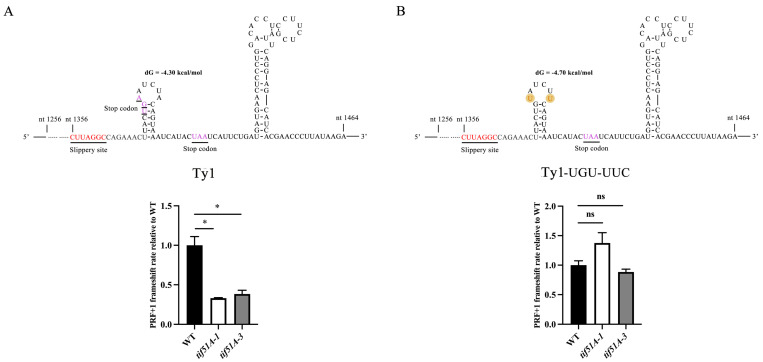
The dependency of *Ty1* +1 PRF on Sc-eIF5A is determined by the distance between the frame 0 stop codon and the slippery sequence. (**A**,**B**) Upper, the sequence and secondary structure of tested frameshifting elements. Lower, the effect of the loss of Sc-eIF5A on the frameshifting of each construct. Red underlined text represents the slippery sequence of *Ty1*. Purple underlined text represents *Ty1* frame 0 stop codons. Error bars denote SD. * *p* < 0.05, ns, not significant (Student’s two-tailed *t* test, *n* = 3, assayed in duplicate).

Given the robust requirement for hypusined Sc-eIF5A for the +1 PRF of reporter transcripts carrying the *Ty1* ribosomal frameshifting element, we next investigated its possible regulatory mechanism. In SARS-CoV-2, the proximity of the ORF1a stop codon to the slippery sequence (18 nucleotides), much less than a ribosomal footprint (approximately 30 nucleotides), confers the dependency of −1 PRF on eIF5A [43]. The distance between the first *Ty1* frame 0 stop codon and the slippery sequence is also 18 nucleotides, much less than a single ribosomal footprint in yeast. So, we investigated whether the proximity of the first *Ty1* frame 0 stop codon to the slippery sequence conferred the dependency of *Ty1* +1 PRF on Sc-eIF5A. We generated a mutant version of the *Ty1* frameshifting element, in which the first frame 0 stop codon was mutated into a sense codon. Two identical nucleotide substitutions left the secondary structure and the free energy of the first stem of the pseudoknot was unaltered, which increased the distance between the slippery sequence and the first frame 0 stop codon to 39 nucleotides, a distance greater than a ribosomal footprint. This mutation did not significantly alter the baseline rate of frameshifting compared with the wild-type frameshifting element (Figure S6). Nevertheless, the dependency of frameshifting on Sc-eIF5A was entirely abolished by the Ty1-UGU-UUC mutation (Figure 7B). Therefore, these results implicate the proximity of the stop codon to the slippery sequence as the key feature that necessitates the dependency of *Ty1* +1 PRF on Sc-eIF5A.

## 3. Discussion

The translation factor eIF5A, originally identified as an initiation factor, was shown to function broadly in translation elongation and termination [28,29]. Recent studies demonstrate that eIF5A and its hypusination is required for the efficient PRF of *OAZ1* mRNA in *S. cerevisiae* [30]. In this report, we employed congenic sets of *tif51A-1* and *tif51A-3* strains expressing either the *Ty1*, *CTS2*, *L-A* or the in-frame reporters to investigate whether the hypusine modification of Sc-eIF5A is vital for the translation of either of the reporters. The results showed that the hypusine modification of Sc-eIF5A is sufficient for +1 PRF of *Ty1*, but its effect on −1 PRF of *L-A* was insufficient. This study represents the first case, indicating that the hypusine modification of eIF5A plays an essential role in the +1 PRF of *Ty1* mRNA in *S. cerevisiae*, indicating that the regulation of +1 PRF by yeast-hypusinated eIF5A is universal. Therefore, these data provide evidence for the in-depth exploration of the +1 PRF mechanism in eukaryotic cells.

Here, the deficiencies of Sc-eIF5A in the *tif51A-1* and *tif51A-3* strains have no effect on the −1 PRF of the *L-A* mRNA, but decrease the −1 PRF of the *CTS2* mRNA (Figure 3B and Figure S1B). Furtherly, the Renilla luciferase of *L-A*, but not *CTS2*, displayed similar activities for all constructs (Figures S2C and S3E,G). Furthermore, a greater decrease in the Fluc than in the Rluc of *CTS2* led to the decrease in the Fluc to Rluc ratios and thus deflated the estimated −1 PRF (Figures S2C,D and S3E–H). These results are similar to previous reports, showing that absolute luciferase activities were reduced in HeLa cells transfected with reporters containing the *CCR5* sequence compared to the HIV-1 control and IFC [40,41]. A greater decrease in Rluc than in Fluc led to an increase in the Fluc to Rluc ratios and thus inflated the estimated −1 PRF [41]. The observed effect is due to the cryptic splicing of the reporter RNA [41], suggesting a possibility that the reporter RNA containing the *CTS2* sequence could be cryptically spliced in *S. cerevisiae*.

Hypusine-modified eIF5A is important for efficient translation termination; its loss of function results in the accumulation of ribosomes at termination codons [43]. Previous studies have shown that the depletion of hypusine-modified eIF5A impairs the −1 PRF of coronavirus SARS-CoV-2 mRNA in human cells [43]. The proximity of the stop codon to the slippery sequence (18 nucleotides), located less than one ribosomal footprint upstream (approximately 30 nucleotides), is the key feature that necessitates efficient termination for frameshifting at the SARS-CoV-2 frameshifting element [43]. Nevertheless, other beta-coronaviruses whose frame 0 stop codons are naturally located farther downstream within the frameshifting element do not require eIF5A for efficient frameshifting [43]. In our studies, the deficiency of hypusine-modified eIF5A in the *tif51A-1* and *tif51A-3* strains also has no effect on the −1 PRF of the *L-A* mRNA in *S. cerevisiae* (Figure 3B). Its frame 0 stop codon was also located farther downstream within the frameshifting element (117 nucleotides), much more than a single ribosomal footprint, suggesting that the L-A virus of yeast also does not require eIF5A for efficient −1 PRF. However, our discovery suggests that Sc-eIF5A is essential for efficient *Ty1* +1 PRF. This dependency is likely due to the proximity of this stop codon to the slippery sequence, located less than one ribosomal footprint upstream. Thus, a trailing ribosome might be sterically inhibited from reaching the slippery sequence if a terminating or post-termination ribosome in the frameshifting element is not rapidly removed, as with the SARS-CoV-2 PRF model [43]. In support of this model, we demonstrated that the relocation of the frame 0 termination codon farther downstream eliminates the requirement for efficient translation termination and ribosome recycling. This result implicates the proximity of the stop codon to the slippery sequence as the key feature that necessitates the efficient clearance of ribosomes from the *Ty1* frame 0 stop codon, promoting the frameshifting of trailing ribosomes. Our finding, therefore, points toward a mechanism in which the stop codon in the first stem of the pseudoknot of the *Ty1* frameshifting element, in concert with the activity of the ribosome recycling machinery, plays a key role in the efficient removal of non-frameshifted ribosomes from the secondary structure and subsequent frameshifting by incoming ribosomes. Hence, we speculate that if a mutant version of the *L-A* frameshifting element, in which the proximity of its frame 0 stop codon to the slippery sequence, located less than one ribosomal footprint upstream, is generated, Sc-eIF5A is also likely to promote PRF at the *L-A* mutant frameshifting element.

Polyamine biosynthesis is under feedback control with the synthesis of multiple enzymes and regulators inhibited by polyamines at the translational level. The high polyamines promote +1 ribosomal frameshifting during the decoding of OAZ in eukaryotes from yeast to humans [23,44,45]. The OAZ binds to ODC, targets it for ubiquitin-independent degradation [46,47], and inhibits putrescine synthesis. In addition, the high polyamines inhibit eIF5A-dependent translation termination on the PS* uORF to repress the synthesis of the ODC antizyme inhibitor (AZIN1), which is a catalytically defective form of ODC that still binds to OAZ [31,32,33]. The down-regulation of the titration of OAZ via AZIN1 enhances OAZ from targeting ODC for degradation, reducing ODC and inhibiting putrescine synthesis. Moreover, the high polyamines inhibit translation termination on the MAGDIS uORF to repress the synthesis of the S-adenosylmethionine decarboxylase (AdoMetDC), and vice versa [48,49,50,51]. In addition to synthesizing polyamines, cells also import polyamines. The high polyamines also inhibit eIF5A-dependent translation termination on the MLLLPS* uORF to repress the synthesis of the polyamine transporter Hol1, and vice versa [34]. Interestingly, polyamines universally promote +1 ribosomal frameshifting efficiency [17,18,52,53].

**Figure 8:**
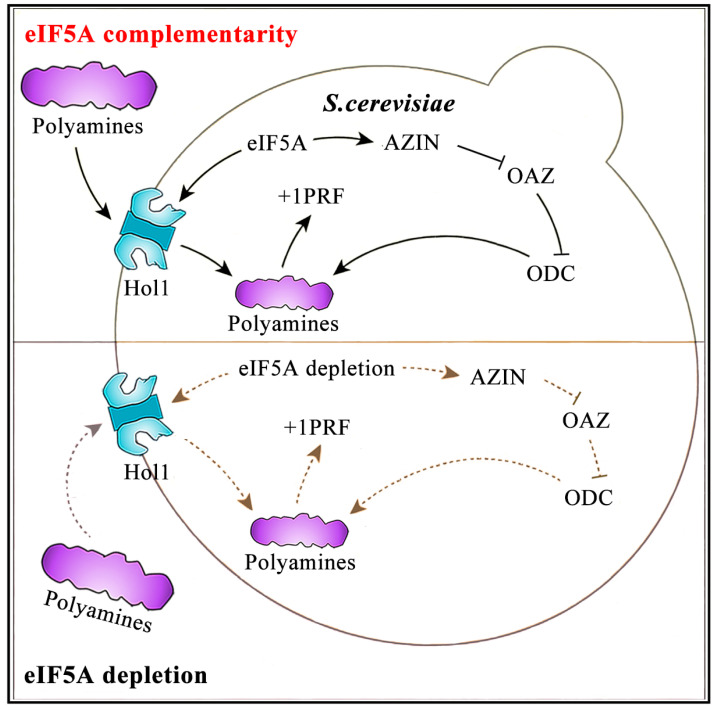
Schematic model of eIF5A and its target genes’ *AZIN1* and *HOL1* functions. Under the condition of eIF5A depletion, AZIN synthesis is suppressed. Down-regulation of the titration of OAZ via AZIN leads to enhanced degradation of OAZ-targeted ODC, reducing ODC, inhibiting putrescine synthesis, and suppressing further +1 PRF. In addition, eIF5A depletion also leads to decreased translation of *HOL1* mRNA, reducing the levels of polyamines and repressing the +1 PRF. Under the condition of eIF5A complementarity, AZIN synthesis is promoted. Titration of OAZ via AZIN prevents OAZ from targeting ODC for degradation, stabilizing ODC, and enhancing putrescine synthesis, promoting further +1 PRF. Moreover, eIF5A complementarity also leads to increased translation of *HOL1* mRNA, increasing the levels of polyamines and promoting the +1 PRF. Solid arrows represent the promotion of downstream proteins by the upstream proteins, whereas dashed arrows are the opposite of solid arrows. And solid lines without arrow represent the inhibition of downstream proteins by the upstream proteins, whereas dashed lines without arrow are the opposite of solid lines without arrow.

Based on these properties of eIF5A and polyamines, we speculated that the hypusinated eIF5A promotes +1 PRF by increasing the putrescine/spermidine ratio. We propose the following model (Figure 8) for the translational control of +1 PRF in yeast. Under the condition of Sc-eIF5A depletion, PPW motif synthesis is inhibited [32]. Ribosomes that initiate at the weak start site of the uCC pause when translating the highly conserved C-terminal PPWxxPS* motif (* = stop codon). The stalled ribosome impedes scanning, and subsequent scanning ribosomes that leaky scan past the uCC start codon without initiating are proposed to form a queue behind the stalled elongating ribosome. Eventually, the queue will extend back to the uCC start codon, poising a scanning ribosome over the uCC start codon for a longer time and enhancing initiation on the uCC. Because ribosomes that translate the uCC do not reinitiate downstream, the increased translation of the uCC represses the synthesis of AZIN [31,32]. The down-regulation of the titration of OAZ via AZIN leads to the enhanced degradation of OAZ-targeted ODC, reducing ODC and inhibiting putrescine synthesis [33,46,47]. And this is thought to inhibit the +1 PRF, and vice versa [17,18,52,53]. On the other hand, Sc-eIF5A depletion can also impair translation termination at a Pro-Ser-stop motif in a conserved up-stream open reading frame on the *HOL1* mRNA to repress HOL1 synthesis [34], which will lead to reduced polyamine synthesis. And this is thought to inhibit the +1 PRF, and vice versa. The regulation of polyamines by Sc-eIF5A is the result of two regulatory pathways. Taken together, eIF5A is a *trans*-regulator of +1 PRF for Ty1 retrotransposon and could function universally in yeast.

## 4. Materials and Methods

### 4.1. Yeast Strains and Growth Conditions

*S. cerevisiae* haploid strains wild-type BY4741 (*MATa his3*Δ*0 leu2*Δ*0 met15*Δ*0 ura3*Δ*0*) and temperature-sensitive eIF5A mutants *tif51A-1* and *tif51A-3* derivatives of the BY4741 strain (gifted from Prof. Paula Alepuz, Universidad de Valencia, Valencia, Spain) cultured in liquid YPD media at 25 °C [39].

### 4.2. The Generation of Ribosomal Frameshift Reporters

To construct the yeast *Ty1*, *CTS2* and *L-A* test reporters, the respective frameshift signals were cloned into the polylinker region of pDB722. The *Ty1* and *CTS2* frameshift signals were amplified from *S. cerevisiae* genome (TransGen Biotech, Beijing, China). The primers are shown in Table 1. The PCR products were digested with Sal I and ligated into pDB722 to create pDB722-Ty1 and pDB722-CTS2, respectively. The coding sequence of *L-A* frameshift signal containing the Sal I restriction site was synthesized and subcloned into pUC57 to create pUC57-L-A. pUC57-L-A was digested with Sal I and ligated into pDB722 to create pDB722-L-A. The synthesized *L-A* sequence is shown in Table 2. The PRF reporter plasmids were transformed into yeast strains, respectively.

### 4.3. The Generation of HYP2 Complementarity Strains

*Sc-HYP2* (NC_001137.3 in GenBank database) was amplified by PCR. Then, *Sc-HYP2* was cloned into pRS315 to create pRS315-Sc-HYP2, which was transformed into *tif51A-1* and *tif51A-3* strains harboring the pDB722 series of plasmids, respectively. The primers are shown in Table 1.

### 4.4. The Generation of HYP2 Hypusination Site Mutant Strains

*Sc-HYP2* hypusination site K51(AAG) was mutated to arginine (AGG) by the directed point mutation method [54], which was named *Sc-HYP2-K51R*. Then, *Sc-HYP2-K51R* was cloned into pRS315 to create pRS315-Sc-HYP2-K51R, which was transformed into *tif51A-1* and *tif51A-3* strains harboring the pDB722 series of plasmids, respectively. The primers are shown in Table 1.

### 4.5. The Inhibition of HYP2 Hypusination in WT Strain by GC7 Treatment

The *WT* strains harboring the pDB722 series of plasmids were grown in 5 mL of YPD medium at 25 °C until they reached approximately 1–2 × 10^7^ cells.mL^−1^. Then, the yeast cells were grown in 5 mL of YPD medium which was added 1 mM GC7 at 37 °C for 5 h.

### 4.6. The Generation of Ty1 Frame 0 Stop Codon Mutant Strains

Two identical nucleotides of the first stem of the pseudoknot of the *Ty1* frameshifting element were substituted by the directed point mutation method [54], which was named *Ty1-UGU-UUC*. Then, *Ty1-UGU-UUC* was cloned into pDB722 to create pDB722-Ty1-UGU-UUC, which was transformed into WT, *tif51A-1* and *tif51A-3* strains, respectively. The primers are shown in Table 1.

### 4.7. The Detection of Polyamines in Yeast Strains by High-Performance Liquid Chromatography (HPLC)

Putrescine, spermidine, and spermine were measured by HPLC using a Supersil ODS2 5 µm column (Elite, Dalian, China). The WT, *tif51A-1*, *tif51A-3, tif51A-1* and *tif51A-3* HYP2 complementarity strains were grown in YPD media (15 mL) at 37 °C for 5 h. Then, yeast cells were collected by centrifugation at 3000× *g* for 5 min. Polyamines were extracted from yeast cell lysate with 5% trichloroacetic acid (TCA) [55], and after centrifugation at 18,000× *g* for 5 min the supernatant was used for HPLC analysis. A reaction mixture containing 400 μL supernatant, 1 mL 2M NaOH and 30 μL benzoyl chloride was incubated at 37 °C for 20 min [56]. The benzoylzed polyamines were extracted with 2 mL of ethyl ether, dried with nitrogen flow, and dissolved in 200 μL of methanol. Aliquots (20 μL) of each sample were injected onto an ODS-C18 column (Elite, 4.6 × 150 mm) and the benzoylzed products were separated at a flow rate of 1 mL/min at 30 °C with a mobile phase of 60/40 (*v*/*v*) methanol/water. The appearance of the benzoylzed products was monitored through changes in absorption at 254 nm.

### 4.8. Analysis of Ribosomal Frameshift Efficiency in tif51A-1, tif51A-3 and HYP2 Complementarity Strains

The *tif51A-1* and *tif51A-3* strains harboring the pDB722 series of plasmids and HYP2 complementarity strains were grown in 5 mL of YPD medium at 25 °C until they reached approximately 1–2 × 10^7^ cells.mL^−1^. Then, the *tif51A-1* and *tif51A-3* strains harboring the pDB722 series of plasmids were grown in uracil^−^ liquid media (15 mL) at 37 °C for 5 h. And the HYP2 complementarity strains were grown in uracil^−^ and leucine^−^ liquid media (15 mL) at 37 °C for 5 h. The yeast cells were collected for subsequent protein analyses by Western blot, mRNA analyses by quantitative real-time PCR and ribosomal frameshift efficiency analyses by Dual-luciferase assays.

### 4.9. Analysis of Ribosomal Frameshift Efficiency in HYP2 Hypusination Site Mutant Strains and WT Strain by GC7 Treatment

The WT strains harboring the pDB722 series of plasmids and the HYP2 hypusination site mutant strains were grown in 5 mL of YPD medium at 25 °C until they reached approximately 1–2 × 10^7^ cells.mL^−1^. Then, the HYP2 hypusination site mutant strains were grown in uracil^−^ and leucine^−^ liquid media (15 mL) at 37 °C for 5 h. And the WT strain by GC7 treatment were grown in YPD liquid media (15 mL) at 37 °C for 5 h. The yeast cells were collected for subsequent protein analyses by Western blot and ribosomal frameshift efficiency analyses by Dual-luciferase assays.

### 4.10. Analysis of Ribosomal Frameshift Efficiency in Ty1 Frame 0 Stop Codon Mutant Strains

The Ty1 frame 0 stop codon mutant strains were grown in 5 mL of uracil^−^ liquid medium at 25 °C until they reached approximately 1–2 × 10^7^ cells.mL^−1^. Then, Ty1 frame 0 stop codon mutant strains were grown in uracil^−^ liquid media (15 mL) at 37 °C for 5 h. The yeast cells were collected for subsequent ribosomal frameshift efficiency analyses by Dual-luciferase assays.

### 4.11. Quantitative Real-Time PCR

Total RNA was isolated from yeast cells using the E.Z.N.A.^®^ Yeast RNA Kit (Omega, Norcross, GA, USA). For cDNA synthesis, the extracted total RNA (2 μg) was treated in a reaction system of Quantscript RT Kit containing 1 μM Oligo (dT) Primer, 20 U TransScript^®^ RT/RI Enzyme Mix, 2 × TS Reaction Mix and gDNA Remover (TRAN, Shanghai, China). Using the resultant cDNA as template and *Sc-actin* as an internal reference gene, *FL* was analyzed by quantitative real-time PCR (qPCR) method. The primers are shown in Table 1. All qPCR were set up using TransStart^®^ Green qPCR SuperMix (TRAN, Shanghai, China) and performed on an Applied Biosystems 7500 Fast Real-Time PCR system (Waltham, MA, USA).

### 4.12. Western Blot

Cells were lysed in RIPA lysis buffer (Beyotime, Jiangsu, China). Samples were frozen and thawed for 1 h followed by centrifugation at 17,000× *g* for 30 min at 4 °C. Cleared protein lysate was denatured with 5× loading buffer for 10 min at 95 °C, and loaded on precast 10% to 15% bis-tris protein gels. Proteins were transferred onto nitrocellulose membranes using the iBLOT 2 system (BIO-RAD, Hercules, CA, USA) following the manufacturer’s protocols. Membranes were blocked with 5% *w*/*v* milk and 0.1% Tween-20 in PBS for 1 h. Then, the membranes were incubated with anti-HA (1:100, Sangon Biotech, Shanghai, China) or anti-α-tubulin (1:400, Sangon Biotech, Shanghai, China) or anti-hypusine (1:400, AtaGenix, Wuhan, China) overnight at 4 °C. The membranes were subsequently incubated with secondary antibody (Abcam, Cambridge, UK) in 5% milk and 0.1% Tween-20 in PBS for 2 h and visualized using Licor Odyssey infrared scanner (Odyssey Clx, Gene Company Limited, Shanghai, China). The optical density of the signals on film was quantified using grayscale measurements in ImageJ software V1.8.0 and converted to fold change. The monoclonal antibody against the hypusine was produced by immunizing rabbits with the synthetic peptide “C-Ahx-STSKTG[hypusine]HGHAKV-amide”.

### 4.13. Dual-Luciferase Assays

The yeast cells were harvested and lysed with 1 mL of ice-cold 1× passive lysis buffer from the Dual-Luciferase^®^ Reporter Assay System (Promega, Madison, WI, USA). Lysates were cleared by centrifugation at 15,000× *g* for 2 min, and the supernatant was assayed for the Renilla luciferase (Rluc) and Firefly luciferase (Fluc) activities, by adding 10 μL of lysate and 10 μL of each reagent, as per the Promega protocol, using Glo MaxTM 20/20 Assay System (Promega, Madison, WI, USA). Frameshift efficiencies were calculated by dividing Fluc values by Rluc values and then dividing the relative ratios by the average Fluc to Rluc ratio of the in-frame control reporter.

## Figures and Tables

**Table 1:** Sequence of primers.

Primers	Sequence (5′ → 3′)
Primers for PCR of the *Ty1* and *CTS2* PRF signals	
Sc-Ty1-F	ACGCGTCGACGAATGTATCGACATCTAATAACTCT
Sc-Ty1-R	ACGCGTCGACGTTCTTATAAGGGTTCGTGAT
Sc-CTS2-F	ACGCGTCGACAAAAAATCAATATTTATCAGTTATGATA
Sc-CTS2-R	ACGCGTCGACGTATTGTTATGTGTCACATATTC
Primers for construction of recombinant pRS315-Sc-HYP2 plasmid for expression in the *tif51A-1* and *tif51A-3* strains	
Sc-HYP2-F	CGGGATCCATGTCTGACGAAGAACATACCT
Sc-HYP2-R	CCCAAGCTTATCGGTTCTAGCAGCTTC
Primers for qPCR	
FL-F	CTAGAGGATGGAACCGCTGGAGAG
FL-R	TCTGCCAACCGAACGGACATTTC
Sc-actin-F	TGGATTCTGAGGTTGCTGCTTTGG
Sc-actin-R	TGGTGTCTTGGTCTACCGACGATAG
Primers for mutation of the frameshifting element of the *Ty1* gene	
M-Sc-Ty1-F	TAGGCCAGAAACTTACTGTATCTTCAGTAAATCATA
M-Sc-Ty1-R	TGATTAGTATGATTTACTGAAGATACAGTAAGTTTCTG
Primers for mutation of the hypusination site of *Sc-HYP2*	
M-Sc-HYP2-F	GTCCACTTCTAAGACTGGTAGGCACGGTCACGCTAA
M-Sc-HYP2-R	TGGACTTTAGCGTGACCGTGCCTACCAGTCTTAGAA

Note: Sequences ‘AAGCTT’, ‘GTCGAC’ and ‘GGATCC’ are the recognition and cleavage sites of restriction endonucleases Hind III, Sal I and BamH I, respectively. ‘T’, ‘A’, ‘AGG’ and ‘CCT’ are the mutation sites of the *Ty1* and *Sc-HYP2* gene, respectively.

**Table 2:** Synthesized gene sequence.

Gene	Sequence (5′ → 3′)
*L-A*	GTCGACGATCAATGCGGGCGAACTTAAGAACTACTGGGGTAGTGTGCGTCGTACTCAGCAGGGTTTAGGAGTGGTAGGTCTTACGATGCCAGCTGTAATGCCTACCGGAGAACCTACAGCTGGCGCTGCCCACGAAGAGTTGATAGAACAGGCGGACAATGTTTTAGTAGAGTAAACGTAATCGAACCCTCACACGGACCCCGCCCTACAAGGTACATACTGCAGACGTCGAC

Note: Sequence ‘GTCGAC’ is the recognition and cleavage site of restriction endonuclease Sal I.

## Data Availability

All relevant data are within the paper and Supplementary Materials. The data used to support the findings of this study are available upon reasonable request.

## Supplementary Materials

The following supporting information can be downloaded at: https://www.mdpi.com/article/10.3390/ijms25031766/s1.

## References

[1] Caliskan N., Peske F., Rodnina M.V. (2015). Changed in translation: mRNA recoding by −1 programmed ribosomal frameshifting. Trends Biochem. Sci..

[2] Choi J., Grosely R., Prabhakar A., Lapointe C.P., Wang J., Puglisi J.D. (2018). How messenger RNA and nascent chain sequences regulate translation elongation. Annu. Rev. Biochem..

[3] Dinman J.D. (2012). Mechanisms and implications of programmed translational frameshifting. Wiley Interdiscip. Rev. RNA.

[4] Jacks T., Varmus H.E. (1985). Expression of the Rous sarcoma virus pol gene by ribosomal frameshifting. Science.

[5] Balasundaram D., Dinman J.D., Wickner R.B., Tabor C.W., Tabor H. (1994). Spermidine deficiency increases +1 ribosomal frameshifting efficiency and inhibits *Ty1* retrotransposition in *Saccharomyces cerevisiae*. Proc. Natl. Acad. Sci. USA.

[6] Dinman J.D., Wickner R.B. (1992). Ribosomal frameshifting efficiency and gag/gag-pol ratio are critical for yeast M1 double-stranded RNA virus propagation. J. Virol..

[7] Dinman J.D., Wickner R.B. (1994). Translational maintenance of frame: Mutants of *Saccharomyces cerevisiae* with altered −1 ribosomal frameshifting efficiencies. Genetics.

[8] Hung M., Patel P., Davis S., Green S.R. (1998). Importance of ribosomal frameshifting for human immunodeficiency virus type 1 particle assembly and replication. J. Virol..

[9] Karacostas V., Wolffe E.J., Nagashima K., Gonda M.A., Moss B. (1993). Overexpression of the HIV-1 gag-pol polyprotein results in intracellular activation of HIV-1 protease and inhibition of assembly and budding of virus-like particles. Virology.

[10] Park J., Morrow C.D. (1991). Overexpression of the gag-pol precursor from human immunodeficiency virus type 1 proviral genomes results in efficient proteolytic processing in the absence of virion production. J. Virol..

[11] Namy O., Rousset J.P., Napthine S., Brierley I. (2004). Reprogrammed genetic decoding in cellular gene expression. Mol. Cell.

[12] Cobucci-Ponzano B., Rossi M., Moracci M. (2012). Translational recoding in archaea. Extremophiles.

[13] Baranov P.V., Gesteland R.F., Atkins J.F. (2002). Recoding: Translational bifurcations in gene expression. Gene.

[14] Kawakami K., Pande S., Faiola B., Moore D.P., Boeke J.D., Farabaugh P.J., Strathern J.N., Nakamura Y., Garfinkel D.J. (1993). A rare tRNA-Arg(CCU) that regulates *Ty1* element ribosomal frameshifting is essential for Ty1 retrotransposition in *Saccharomyces cerevisiae*. Genetics.

[15] Atkins J.F., Björk G.R. (2009). A gripping tale of ribosomal frameshifting: Extragenic suppressors of frameshift mutations spotlight P-site realignment. Microbiol. Mol. Biol. Rev..

[16] Tuohy T.M., Thompson S., Gesteland R.F., Atkins J.F. (1992). Seven, eight and nine-membered anticodon loop mutants of tRNA(2Arg) which cause +1 frameshifting. Tolerance of DHU arm and other secondary mutations. J. Mol. Biol..

[17] Higashi K., Kashiwagi K., Taniguchi S., Terui Y., Yamamoto K., Ishihama A., Igarashi K. (2006). Enhancement of +1 frameshift by polyamines during translation of polypeptide release factor 2 in *Escherichia coli*. J. Biol. Chem..

[18] Balasundaram D., Dinman J.D., Tabor C.W., Tabor H. (1994). SPE1 and SPE2: Two essential genes in the biosynthesis of polyamines that modulate +1 ribosomal frameshifting in *Saccharomyces cerevisiae*. J. Bacteriol..

[19] Gupta P., Kannan K., Mankin A.S., Vázquez-Laslop N. (2013). Regulation of gene expression by macrolide-induced ribosomal frameshifting. Mol. Cell.

[20] Harger J.W., Meskauskas A., Dinman J.D. (2002). An “integrated model” of programmed ribosomal frameshifting. Trends Biochem. Sci..

[21] Napthine S., Ling R., Finch L.K., Jones J.D., Bell S., Brierley I., Firth A.E. (2017). Protein-directed ribosomal frameshifting temporally regulates gene expression. Nat. Commun..

[22] Dinman J.D. (1995). Ribosomal frameshifting in yeast viruses. Yeast.

[23] Palanimurugan R., Scheel H., Hofmann K., Dohmen R.J. (2004). Polyamines regulate their synthesis by inducing expression and blocking degradation of ODC antizyme. EMBO J..

[24] Dever T.E., Gutierrez E., Shin B.S. (2014). The hypusine-containing translation factor eIF5A. Crit. Rev. Biochem. Mol. Biol..

[25] Park M.H. (2006). The post-translational synthesis of a polyamine-derived amino acid, hypusine, in the eukaryotic translation initiation factor 5A (eIF5A). J. Biochem..

[26] Park M.H., Wolff E.C. (2018). Hypusine, a polyamine-derived amino acid critical for eukaryotic translation. J. Biol. Chem..

[27] Gutierrez E., Shin B.S., Woolstenhulme C.J., Kim J.R., Saini P., Buskirk A.R., Dever T.E. (2013). eIF5A promotes translation of polyproline motifs. Mol. Cell.

[28] Schuller A.P., Wu C.C., Dever T.E., Buskirk A.R., Green R. (2017). eIF5A functions globally in translation elongation and termination. Mol. Cell.

[29] Pelechano V., Alepuz P. (2017). eIF5A facilitates translation termination globally and promotes the elongation of many nonpolyproline-specific tripeptide sequences. Nucleic Acids Res..

[30] Halwas K., Döring L.M., Oehlert F.V., Dohmen R.J. (2022). Hypusinated eIF5A promotes ribosomal frameshifting during decoding of ODC antizyme mRNA in *Saccharomyces cerevisiae*. Int. J. Mol. Sci..

[31] Ivanov I.P., Loughran G., Atkins J.F. (2008). uORFs with unusual translational start codons autoregulate expression of eukaryotic ornithine decarboxylase homologs. Proc. Natl. Acad. Sci. USA.

[32] Ivanov I.P., Shin B.S., Loughran G., Tzani I., Young-Baird S.K., Cao C., Atkins J.F., Dever T.E. (2018). Polyamine control of translation elongation regulates start site selection on antizyme inhibitor mRNA via ribosome queuing. Mol. Cell.

[33] Murakami Y., Ichiba T., Matsufuji S., Hayashi S. (1996). Cloning of antizyme inhibitor, a highly homologous protein to ornithine decarboxylase. J. Biol. Chem..

[34] Vindu A., Shin B.S., Choi K., Christenson E.T., Ivanov I.P., Cao C., Banerjee A., Dever T.E. (2021). Translational autoregulation of the *S. cerevisiae* high-affinity polyamine transporter Hol1. Mol. Cell.

[35] Dinman J.D., Icho T., Wickner R.B. (1991). A -1 ribosomal frameshift in a double-stranded RNA virus of yeast forms a gag-pol fusion protein. Proc. Natl. Acad. Sci. USA.

[36] Tu C., Tzeng T.H., Bruenn J.A. (1992). Ribosomal movement impeded at a pseudoknot required for frameshifting. Proc. Natl. Acad. Sci. USA.

[37] Farabaugh P.J. (1996). Programmed translational frameshifting. Microbiol. Rev..

[38] Jacobs J.L., Belew A.T., Rakauskaite R., Dinman J.D. (2007). Identification of functional, endogenous programmed -1 ribosomal frameshift signals in the genome of *Saccharomyces cerevisiae*. Nucleic Acids Res..

[39] Li Z., Vizeacoumar F.J., Bahr S., Li J., Warringer J., Vizeacoumar F.S., Min R., Vandersluis B., Bellay J., Devit M. (2011). Systematic exploration of essential yeast gene function with temperature-sensitive mutants. Nat. Biotechnol..

[40] Belew A.T., Meskauskas A., Musalgaonkar S., Advani V.M., Sulima S.O., Kasprzak W.K., Shapiro B.A., Dinman J.D. (2014). Ribosomal frameshifting in the *CCR5* mRNA is regulated by miRNAs and the NMD pathway. Nature.

[41] Khan Y.A., Loughran G., Steckelberg A.L., Brown K., Kiniry S.J., Stewart H., Baranov P.V., Kieft J.S., Firth A.E., Atkins J.F. (2022). Evaluating ribosomal frameshifting in *CCR5* mRNA decoding. Nature.

[42] Xiao Y., Li J., Wang R., Fan Y., Han X., Fu Y., Alepuz P., Wang W., Liang A. (2024). eIF5A promotes +1 programmed ribosomal frameshifting in *Euplotes octocarinatus*. Int. J. Biol. Macromol..

[43] Rehfeld F., Eitson J.L., Ohlson M.B., Chang T.C., Schoggins J.W., Mendell J.T. (2023). CRISPR screening reveals a dependency on ribosome recycling for efficient SARS-CoV-2 programmed ribosomal frameshifting and viral replication. Cell Rep..

[44] Matsufuji S., Matsufuji T., Miyazaki Y., Murakami Y., Atkins J.F., Gesteland R.F., Hayashi S. (1995). Autoregulatory frameshifting in decoding mammalian ornithine decarboxylase antizyme. Cell.

[45] Ivanov I.P., Atkins J.F. (2007). Ribosomal frameshifting in decoding antizyme mRNAs from yeast and protists to humans: Close to 300 cases reveal remarkable diversity despite underlying conservation. Nucleic Acids Res..

[46] Heller J.S., Canellakis E.S. (1981). Cellular control of ornithine decarboxylase activity by its antizyme. J. Cell Physiol..

[47] Murakami Y., Matsufuji S., Kameji T., Hayashi S., Igarashi K., Tamura T., Tanaka K., Ichihara A. (1992). Ornithine decarboxylase is degraded by the 26S proteasome without ubiquitination. Nature.

[48] Ruan H., Shantz L.M., Pegg A.E., Morris D.R. (1996). The upstream open reading frame of the mRNA encoding S-adenosylmethionine decarboxylase is a polyamine-responsive translational control element. J. Biol. Chem..

[49] Law G.L., Raney A., Heusner C., Morris D.R. (2001). Polyamine regulation of ribosome pausing at the upstream open reading frame of S-adenosylmethionine decarboxylase. J. Biol. Chem..

[50] Raney A., Law G.L., Mize G.J., Morris D.R. (2002). Regulated translation termination at the upstream open reading frame in S-adenosylmethionine decarboxylase mRNA. J. Biol. Chem..

[51] Pegg A.E. (2009). Mammalian polyamine metabolism and function. IUBMB Life.

[52] Kurian L., Palanimurugan R., Godderz D., Dohmen R.J. (2011). Polyamine sensing by nascent ornithine decarboxylase antizyme stimulates decoding of its mRNA. Nature.

[53] Yordanova M.M., Wu C., Andreev D.E., Sachs M.S., Atkins J.F. (2015). A nascent peptide signal responsive to endogenous levels of polyamines acts to stimulate regulatory frameshifting on antizyme mRNA. J. Biol. Chem..

[54] Saini P., Eyler D.E., Green R., Dever T.E. (2009). Hypusine-containing protein eIF5A promotes translation elongation. Nature.

[55] Kera K., Nagayama T., Nanatani K., Saeki-Yamoto C., Tominaga A., Souma S., Miura N., Takeda K., Kayamori S., Ando E. (2018). Reduction of spermidine content resulting from inactivation of two arginine decarboxylases increases biofilm formation in *Synechocystis* sp. strain PCC 6803. J. Bacteriol..

[56] Ozdestan O., Uren A. (2009). A method for benzoyl chloride derivatization of biogenic amines for high performance liquid chromatography. Talanta.

