# Supplementary material for: The Deficiency of Hypusinated eIF5A Decreases the Putrescine/Spermidine Ratio and Inhibits +1 Programmed Ribosomal Frameshifting during the Translation of Ty1 Retrotransposon in Saccharomyces cerevisiae
Source: Int J Mol Sci. 2024 Feb 1;25(3):1766. doi: 10.3390/ijms25031766 (PMC10855120; doi:10.3390/ijms25031766)
Supplement: Supplementary file 1 [file ijms-25-01766-s001.zip › ijms-2771793-supplementary.pdf]

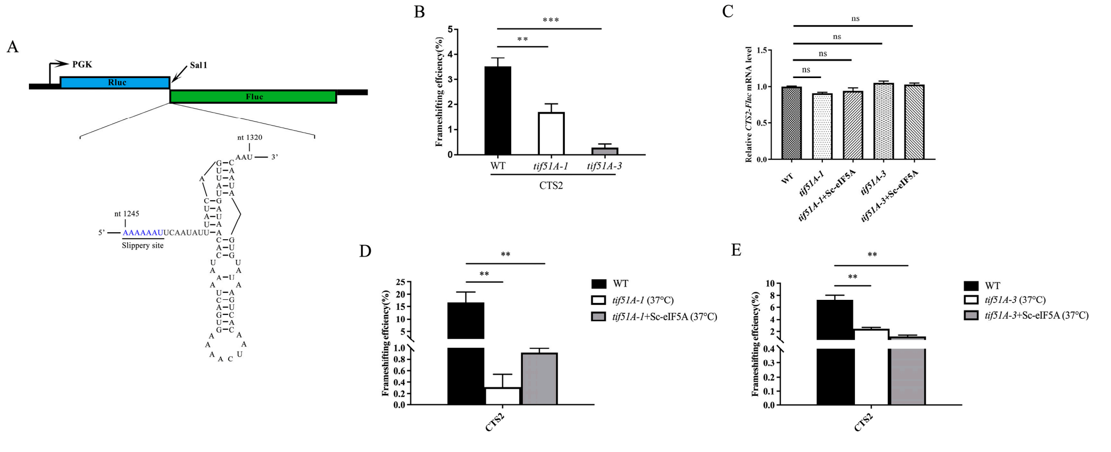

Figure S1. Detection of the influence of Sc-eIF5A on *CTS2* -1 PRF. (A) Secondary structural representation of the *CTS2* -1 PRF signal. Graphical representation of the pDB722-CTS2. Blue underlined text represents the slippery sequence of *CTS2*. (B) Dual-luciferase reporter plasmids containing Fluc and Rluc coding regions separated by the +1 PRF signal from the -1 PRF signal from the yeast *CTS2* chitinase-encoding gene, or the 0-frame control were introduced into WT, *tif51A-1* and *tif51A-3* strains. (C) Relative *CTS2-Fluc* mRNA levels were determined by qPCR and first normalized to actin mRNA. Then, for each panel, measurements were normalized to WT samples. (D) *tif51A-1* and Sc-eIF5A-complemented *tif51A-1* strains were grown at 37 °C for 5 h. (E) *tif51A-3* and Sc-eIF5A-complemented *tif51A-3* strains were grown at 37 °C for 5 h. Dual-luciferase reporter plasmids containing Fluc and Rluc coding regions separated by the -1 PRF signal from the yeast *CTS2* chitinase-encoding gene, or the 0-frame control were introduced into WT, *tif51A-1* and *tif51A-3* mutant strains. PRF efficiencies (%) were calculated by dividing the ratio of Fluc to Rluc obtained with the reporter versus the 0-frame control plasmid. Error bars denote SD. **p < 0.01, ***p < 0.001, ns, not significant (Student's two-tailed t test, *n* = 3, assayed in duplicate).

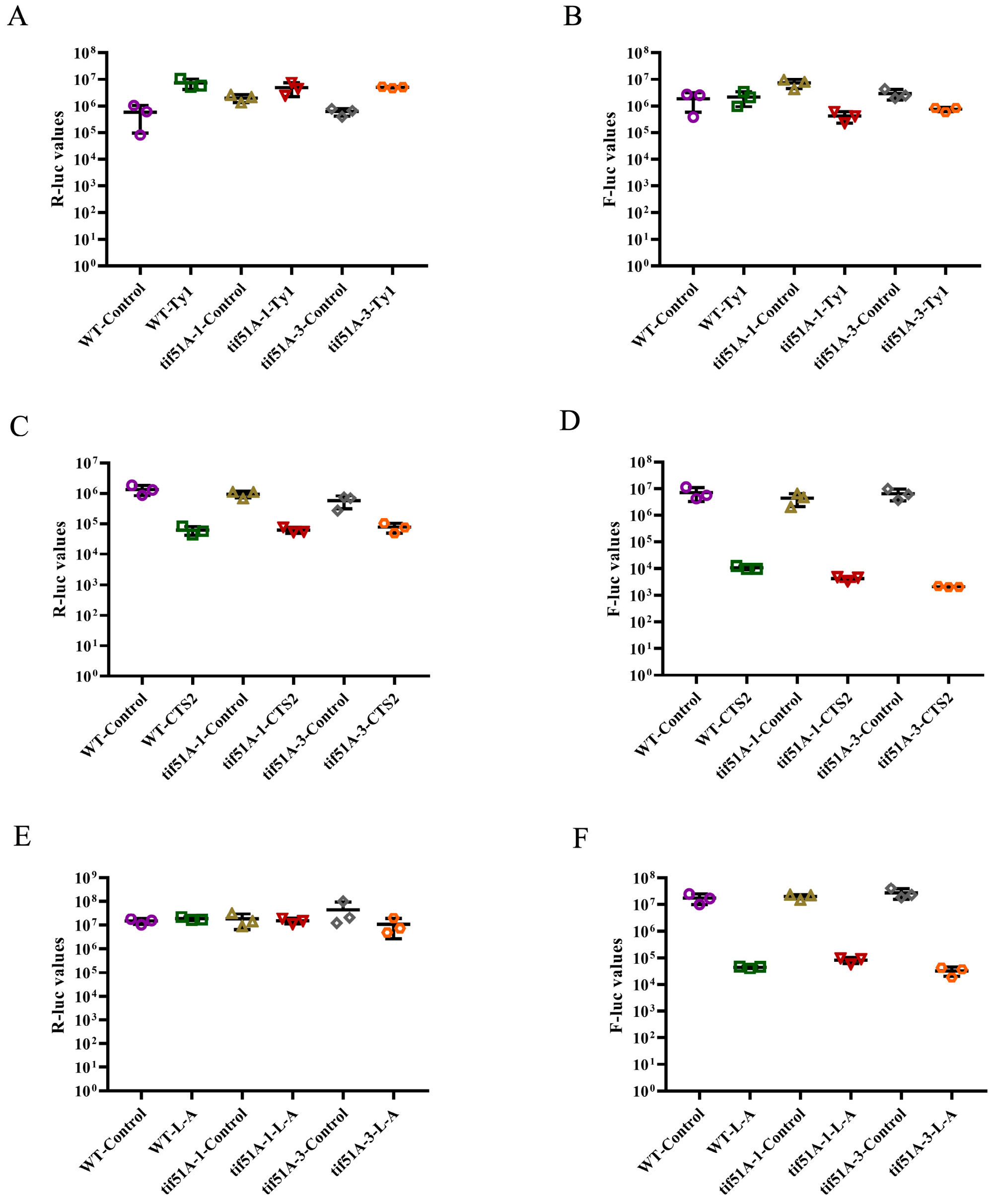

Figure S2. Luciferase values for each experiment, related to Figure 3 and Figure S1B. (A) Absolute Rluc values for *Ty1* DNA transfection experiments performed in WT, *tif51A-1* and *tif51A-3* strains. (B) Absolute Fluc values for *Ty1* DNA transfection experiments performed in WT, *tif51A-1* and *tif51A-3* strains. (C) Absolute Rluc values for *CTS2* DNA transfection experiments performed in WT, *tif51A-1* and *tif51A-3* strains. (D) Absolute Fluc values for *CTS2* DNA transfection experiments performed in WT, *tif51A-1* and *tif51A-3* strains. (E) Absolute Rluc values for *L-A* DNA transfection experiments performed in WT, *tif51A-1* and *tif51A-3* strains. (F) Absolute Fluc values for *L-A* DNA transfection experiments performed in WT, *tif51A-1* and *tif51A-3* strains. Error bars denote SD. And individual replicates are plotted with a symbol. Different symbols represent different groups of replicates (n = 3, assayed in duplicate).

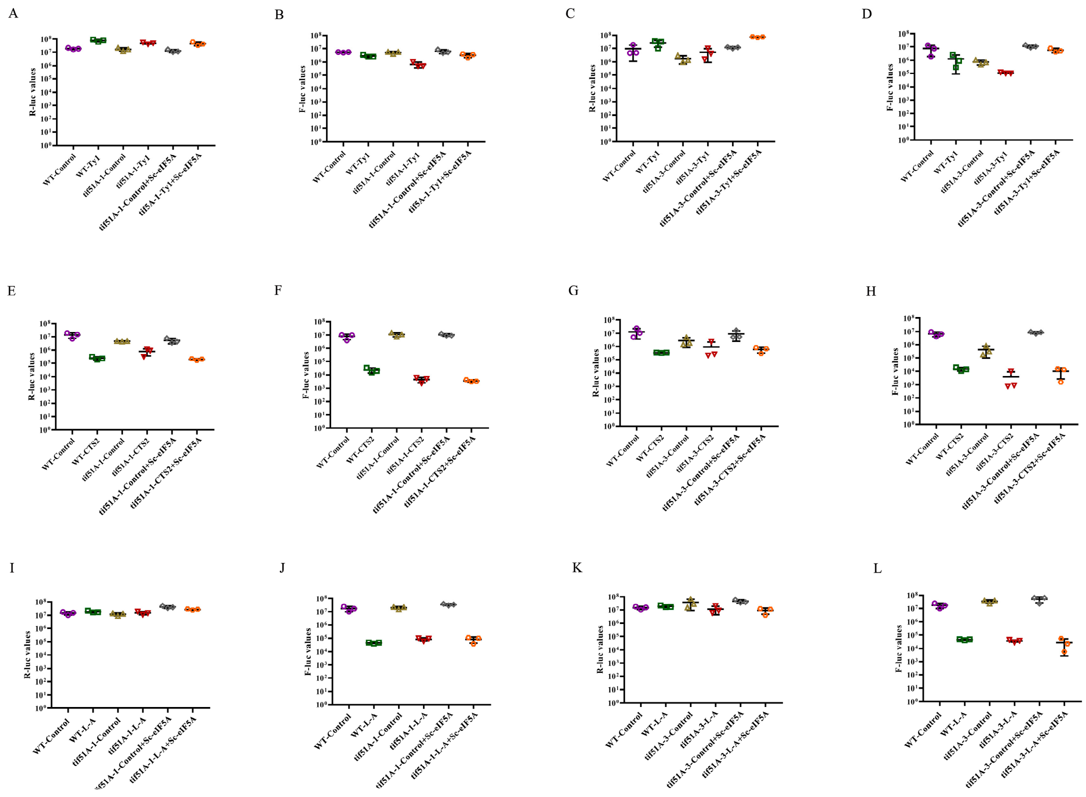

Figure S3. Luciferase values for each experiment, related to Figure 5 and Figure S1D, E. (A) Absolute Rluc values for *Ty1* DNA transfection experiments performed in WT, tif51A-1 and Sc-eIF5A-complemented tif51A-1 strains. (B) Absolute Fluc values for *Ty1* DNA transfection experiments performed in WT, tif51A-1 and Sc-eIF5A-complemented tif51A-1 strains. (C) Absolute Rluc values for *Ty1* DNA transfection experiments performed in WT, tif51A-3 and Sc-eIF5A-complemented tif51A-3 strains. (D) Absolute Fluc values for *Ty1* DNA transfection experiments performed in WT, *tif51A-3* and Sc-eIF5A-complemented *tif51A-3* strains. (E) Absolute Rluc values for *CTS2* DNA transfection experiments performed in WT, *tif51A-1* and Sc-eIF5A-complemented *tif51A-1* strains. (F) Absolute Fluc values for *CTS2* DNA transfection experiments performed in WT, *tif51A-1* and Sc-eIF5A-complemented *tif51A-1* strains. (G) Absolute Rluc values for *CTS2* DNA transfection experiments performed in WT, *tif51A-3* and Sc-eIF5A-complemented *tif51A-3* strains. (H) Absolute Fluc values for *CTS2* DNA transfection experiments performed in WT, *tif51A-3* and Sc-eIF5A-complemented *tif51A-3* strains. (I) Absolute Rluc values for *L-A* DNA transfection experiments performed in WT, *tif51A-1* and Sc-eIF5A-complemented *tif51A-1* strains. (J) Absolute Fluc values for *L-A* DNA transfection experiments performed in WT, *tif51A-1* and Sc-eIF5A-complemented *tif51A-1* strains. (K) Absolute Rluc values for *L-A* DNA transfection experiments performed in WT, *tif51A-3* and Sc-eIF5A-complemented *tif51A-3* strains. (L) Absolute Fluc values for *L-A* DNA transfection experiments performed in WT, *tif51A-3* and Sc-eIF5A-complemented *tif51A-3* strains. Error bars denote SD. And individual replicates are plotted with a symbol. Different symbols represent different groups of replicates (n = 3, assayed in duplicate).

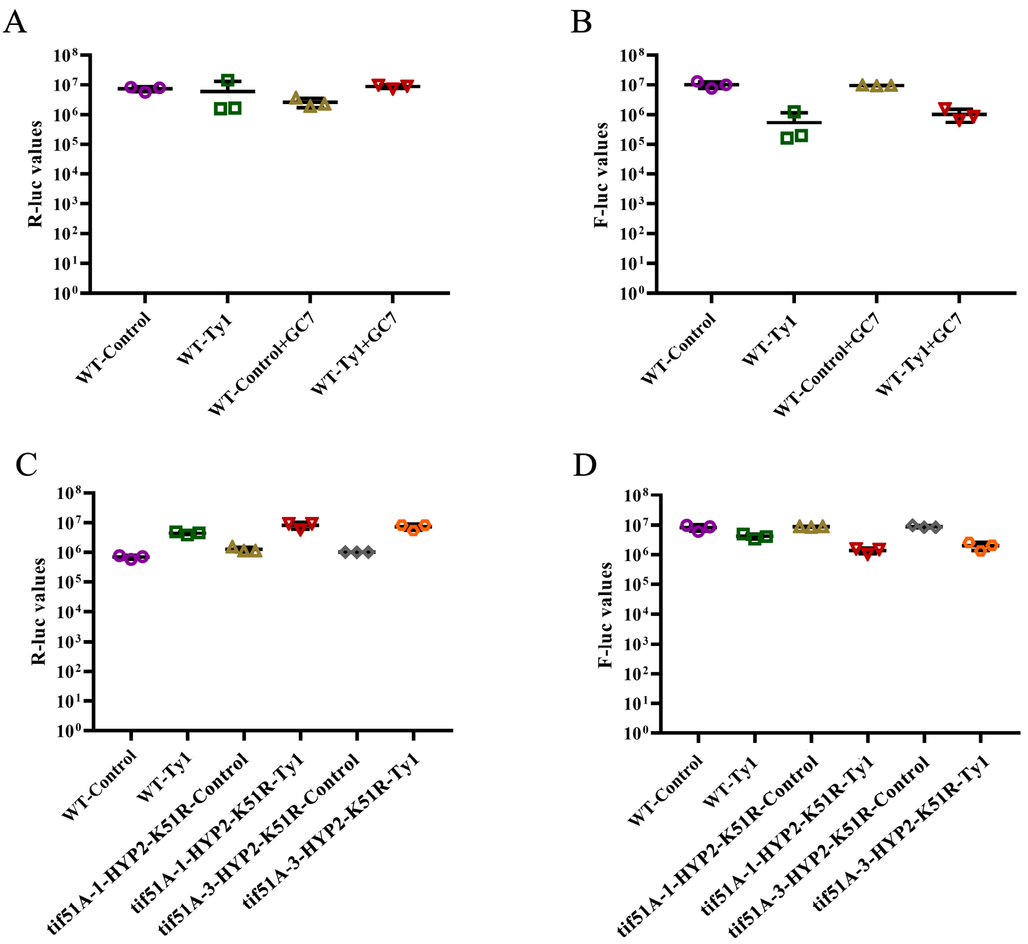

Figure S4. Luciferase values for each experiment, related to Figure 6B and D. (A) Absolute Rluc values for *Ty1* DNA transfection experiments performed in WT and GC7-treated WT strains. (B) Absolute Fluc values for *Ty1* DNA transfection experiments performed in WT and GC7-treated WT strains. (C) Absolute Rluc values for *Ty1* DNA transfection experiments performed in WT, tif51A-1-HYP2-K51R and tif51A-3-HYP2-K51R strains. (D) Absolute Fluc values for *Ty1* DNA transfection experiments performed in WT, tif51A-1-HYP2-K51R and tif51A-3-HYP2-K51R strains. Error bars denote SD. And individual replicates are plotted with a symbol. Different symbols represent different groups of replicates (n = 3, assayed in duplicate).

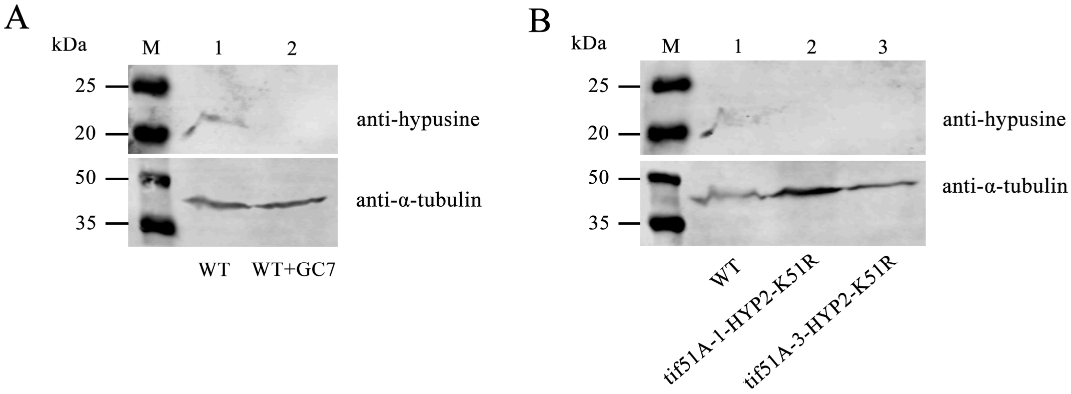

Figure S5. Western blot analysis of the hypusine levels of Sc-eIF5A, replicates of Figure 6A and C. (A) WT and GC7-treated WT strains were grown at 37 °C for 5 h (B) WT, tif51A-1-HYP2-K51R and tif51A-3-HYP2-K51R strains were grown at 37 °C for 5 h.

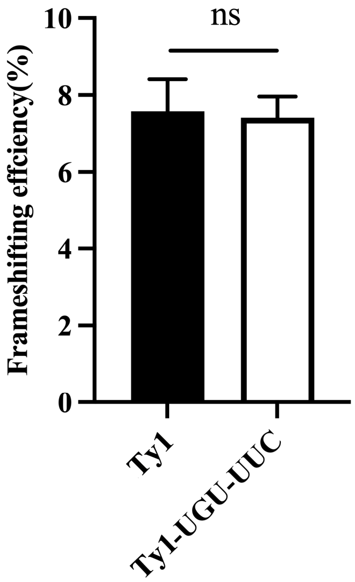

Figure S6. Ty1-UGU-UUC mutation did not significantly alter the baseline rate of frameshifting compared with the wild-type Ty1 frameshifting element. Basal frameshifting rate of reporter constructs harboring the indicated Ty1 or Ty1-UGU-UUC frameshifting element. PRF efficiencies (%) were calculated by dividing the ratio of Fluc to Rluc obtained with the reporter versus the 0-frame control plasmid. Error bars denote SD. ns, not significant (Student's two-tailed t test, n = 3, assayed in duplicate).
